# Molecular basis of phenotypic plasticity in a marine ciliate

**DOI:** 10.1093/ismejo/wrae136

**Published:** 2024-07-17

**Authors:** Jiao Pan, Yaohai Wang, Chao Li, Simo Zhang, Zhiqiang Ye, Jiahao Ni, Haichao Li, Yichen Li, Hongwei Yue, Chenchen Ruan, Dange Zhao, Yujian Jiang, Xiaolin Wu, Xiaopeng Shen, Rebecca A Zufall, Yu Zhang, Weiyi Li, Michael Lynch, Hongan Long

**Affiliations:** Key Laboratory of Evolution and Marine Biodiversity (Ministry of Education), Institute of Evolution and Marine Biodiversity, Ocean University of China, Qingdao, Shandong 266003, China; Laboratory for Marine Biology and Biotechnology, Qingdao Marine Science and Technology Center, Qingdao, Shandong 266237, China; Key Laboratory of Evolution and Marine Biodiversity (Ministry of Education), Institute of Evolution and Marine Biodiversity, Ocean University of China, Qingdao, Shandong 266003, China; Key Laboratory of Evolution and Marine Biodiversity (Ministry of Education), Institute of Evolution and Marine Biodiversity, Ocean University of China, Qingdao, Shandong 266003, China; Department of Biology, Indiana University, Bloomington, IN 47405, United States; School of Life Sciences, Central China Normal University, Wuhan, Hubei 430079, China; Key Laboratory of Evolution and Marine Biodiversity (Ministry of Education), Institute of Evolution and Marine Biodiversity, Ocean University of China, Qingdao, Shandong 266003, China; Key Laboratory of Evolution and Marine Biodiversity (Ministry of Education), Institute of Evolution and Marine Biodiversity, Ocean University of China, Qingdao, Shandong 266003, China; Key Laboratory of Evolution and Marine Biodiversity (Ministry of Education), Institute of Evolution and Marine Biodiversity, Ocean University of China, Qingdao, Shandong 266003, China; Key Laboratory of Evolution and Marine Biodiversity (Ministry of Education), Institute of Evolution and Marine Biodiversity, Ocean University of China, Qingdao, Shandong 266003, China; Key Laboratory of Evolution and Marine Biodiversity (Ministry of Education), Institute of Evolution and Marine Biodiversity, Ocean University of China, Qingdao, Shandong 266003, China; Key Laboratory of Evolution and Marine Biodiversity (Ministry of Education), Institute of Evolution and Marine Biodiversity, Ocean University of China, Qingdao, Shandong 266003, China; Key Laboratory of Evolution and Marine Biodiversity (Ministry of Education), Institute of Evolution and Marine Biodiversity, Ocean University of China, Qingdao, Shandong 266003, China; Key Laboratory of Evolution and Marine Biodiversity (Ministry of Education), Institute of Evolution and Marine Biodiversity, Ocean University of China, Qingdao, Shandong 266003, China; College of Life Sciences, Anhui Normal University, Wuhu, Anhui 241000, China; Department of Biology and Biochemistry, University of Houston, Houston, TX 77204, United States; School of Mathematics Science, Ocean University of China, Qingdao, Shandong Province 266000, China; Department of Genetics, Stanford University School of Medicine, Stanford, CA 94305, United States; Biodesign Center for Mechanisms of Evolution, Arizona State University, Tempe, AZ 85287, United States; Key Laboratory of Evolution and Marine Biodiversity (Ministry of Education), Institute of Evolution and Marine Biodiversity, Ocean University of China, Qingdao, Shandong 266003, China; Laboratory for Marine Biology and Biotechnology, Qingdao Marine Science and Technology Center, Qingdao, Shandong 266237, China

**Keywords:** microbial evolution, life history, macronucleus, protist

## Abstract

Phenotypic plasticity, which involves phenotypic transformation in the absence of genetic change, may serve as a strategy for organisms to survive in complex and highly fluctuating environments. However, its reaction norm, molecular basis, and evolution remain unclear in most organisms, especially microbial eukaryotes. In this study, we explored these questions by investigating the reaction norm, regulation, and evolution of phenotypic plasticity in the cosmopolitan marine free-living ciliates *Glauconema* spp., which undergo significant phenotypic changes in response to food shortages. This study led to the *de novo* assembly of macronuclear genomes using long-read sequencing, identified hundreds of differentially expressed genes associated with phenotypic plasticity in different life stages, validated the function of two of these genes, and revealed that the reaction norm of body shape in response to food density follows a power-law distribution. Purifying selection may be the dominant evolutionary force acting on the genes associated with phenotypic plasticity, and the overall data support the hypothesis that phenotypic plasticity is a trait maintained by natural selection. This study provides novel insight into the developmental genetics of phenotypic plasticity in non-model unicellular eukaryotes and sheds light on the complexity and long evolutionary history of this important survival strategy.

## Introduction

Phenotypic plasticity refers to the ability of organisms to produce more than one phenotype from a single genotype, generally reflecting a physiological response to different environmental conditions [1–4]. Such strategies can be crucial for organisms living in highly variable or unpredictable environments, allowing them to adjust to changing conditions and sometimes promoting population persistence in the face of harsh conditions [5–8]. Reaction norms describe and quantify the phenotypic responses for a given genotype to different levels of environmental factors [9]. Although the costs and benefits of phenotypic plasticity have been well studied in ecology and evolution, they are still not fully explored empirically, in terms of reaction norm, regulation, or evolution [10–15].

Although it is generally accepted that sessile organisms, such as plants, may rely more heavily on plasticity than motile organisms that can move away from unfavorable conditions, in animals there are also numerous examples of such, e.g. plasticity in crab claw sizes in response to the hardness of prey, dung beetles’ male mating strategies, and so on [16–18]. Studying their phenotypic plasticity could deepen our understanding of how organisms and ecosystems respond to climate change [19–21]. It is also necessary to integrate ecological mechanisms to explore the influence of global change on phenotypic plasticity at the population level [22]. An organism’s adaptive potential is strongly influenced not only by the performance of each independent stage but also by the differences in form or function and the distinct ecological niches of these stages [23, 24]. Moreover, the phenomenon is not limited to individual organisms, but can also occur at the cellular level within a multicellular organism, where it can be associated with diseases [25]. Given its broad ecological, evolutionary, and medical implications, the study of phenotypic plasticity is thus of great significance [18, 26–29]. Previous studies on phenotypic plasticity focused on animals and plants and related theoretical modeling and phenotyping experiments [18, 30–36], with a more recent focus on functional genomics approaches [37].

Unicellular eukaryotes encompass more phylogenetic diversity, numerical abundance, and global biomass than animals and plants [38]. They are major members of the microbial food loop, a complex system of interactions between microorganisms that recycles and transforms nutrients in aquatic ecosystems and plays a crucial role in sustaining the productivity and diversity of these ecosystems [39, 40]. Phenotypic plasticity has also been frequently observed in unicellular eukaryotes [41–43]. For example, some ciliated protozoa (*Euplotes versatilis*, *Tetrahymena vorax*) can undergo unequal fissions to generate giant cells that are predatory or cannibalistic in response to variation of food availability, and the unicellular green alga *Chlamydomonas reinhardtii* can form multicellular structures when predators are present [44–46]. Studies have shown that phenotypic plasticity can strongly affect trait variation along thermal gradients, when cells are challenged by climate change and ocean acidification, and phenotypic plasticity may allow organisms to maintain homeostasis and avoid extinction [47–49]. Thus, besides addressing the aforementioned questions, understanding the mechanisms and limits of phenotypic plasticity is critical for predicting the impacts of climate change on unicellular eukaryotes and their roles in ecosystems. Also, investigating the evolution of phenotypic plasticity in unicellular eukaryotes can provide insights into the origins of multicellularity and the development of complex life histories [45, 50].

Ciliates comprise a diverse group of microbial eukaryotes that are abundant in aquatic ecosystems and soils [51–53]. These fascinating organisms have unique nuclear dimorphism, possessing a germline micronucleus, and a somatic macronucleus in the same cytoplasm. Ciliates have long been a focus for microbial ecologists, who study how these organisms respond to climate change in order to provide predictive tools [54–57]. The study of phenotypic plasticity can reveal how organisms behave in various ecological environments and may improve the accuracy of the predictions. Although some cases of phenotypic plasticity in ciliates were also reported [45, 53, 58, 59], previous investigations have rarely studied either the reaction norm or the molecular basis that underlies these transformations.

*Glauconema* is a genus of free-living and cosmopolitan ciliates, widely found in global coastal waters [60–62]. Despite its potential as a research model for phenotypic plasticity, these bacteria-grazers have received little attention from the standpoint of genetics, genomics, or phenotypic variation. Specifically, in response to food scarcity, the broad-bean-shaped, and slowly-moving cells in vegetative growth (trophonts) transform into fusiform and fast swimmers (tomites), which hover in the water layer and suddenly dash. In some species, cells further transform into resting cysts if food bacteria are in lower density [60–62]. Due to the influence of geographical location and global environmental changes, some non-marine ciliates encounter high temperatures and high salinity. In response, they have evolved adaptive mechanisms, which have been partially explored in previous studies [52, 53, 58, 59, 63, 64]. The environmental stresses experienced by *Glauconema* in the ocean, encompassing factors such as temperature, salinity, hypoxia, air exposure, and anthropogenic pollutants, parallel that of other coastal organisms, such as the heterotrophic flagellate *Oxyrrhis marina*, the growth rate and cell volume of which show great plasticity upon temperature or food concentration change [65]. Thus, investigations into the phenotypic plasticity of *Glauconema* may provide broader insights into the genetic regulation and evolution of marine organisms.

In this study, we explore the phenotypic plasticity of *Glauconema* spp. collected from the coastal waters of northern (*G*. sp1 LHA0827) and southern China (*G*. sp2 LJL43), deriving the reaction norms of the body shape vs. food bacteria density, analyzing critical genes/pathways based on differential gene expression from low-input RNAseq combined with RT-qPCR and RNAi techniques, and *de novo* assembling and annotating their macronuclear genomes. By filling in the gaps in our understanding of the basic genome biology of the genus as well as revealing aspects of phenotypic plasticity, this study thus provides a framework for further understanding the genetics and evolution of this globally important group of eukaryotic microbes.

## Materials and methods

### Species isolation, culture, and identification

*Glauconema* sp1 LHA0827 and *G*. sp2 LJL43 were collected from the coastal water of Qingdao, Shandong Province (36.06° N, 120.37° E; 27°C; pH = 8.01; 27 August 2017) and Danzhou, Hainan Province (19.67° N, 109.09° E; 26.3°C; pH = 8.16; 22 June 2019), China, respectively. Strain cultures are available upon request at the Institute of Evolution and Marine Biodiversity, Ocean University of China (https://iemb.ouc.edu.cn/labstrains/list.htm). Each culture was established from one single cell, which was rinsed by serial dilution in autoclaved seawater with 10 μg/ml Penicillin-Streptomycin-Amphotericin B (Cat. No.: 03-033-1B; Biological Industries). Then we fed the cells with the food bacteria *Pseudoalteromonas* sp. LC2018020214 suspended in autoclaved seawater at OD_600_ = 0.3 and 25°C. The bacterium was isolated from the same sample as *G*. sp1 LHA0827 and cultured with marine LB broth on a 200 rpm shaker at 25°C, using a lab-made recipe [66, 67].

Randomly selected living cells were observed using bright field and differential interference contrast microscopy at 1000× magnification (Nikon Eclipse Ni-U). The infraciliature was revealed by the protargol staining method [68]. The protargol powder was made following a lab-developed recipe [69]. Hoechst 33342 staining for 30 min was used to reveal the nuclear apparatus using the fluorescent module of the microscope (Nikon Eclipse Ni-U). The immuofluoresence (IF) staining was done on trophonts cultured for 48 h after inoculation following Greer *et al.* [70]. Briefly, cells were incubated for 2 h at room temperature with the primary antibody—Monoclonal Anti-α-Tubulin antibody produced in mouse (Cat. No.: T5168; 1:10 000; Sigma). Then, we incubated the cells with the secondary antibody (Goat anti-rabbit IgG (H + L); Cat. No.: A-21428; 1:2000; Sigma) for 1 h at room temperature. The photomicrographs were taken using a Nikon Y-TV55 microscope and a Nikon DS-Ri2 camera. Finally, the nucleus was displayed with DAPI staining (ProLong gold antifade mountant with DAPI; Cat. No.: P36935; Invitrogen) for 2 min. We identified species by the morphological features and 18S rRNA gene sequences. The PCR primers used for amplification were EukA (5′-AACCTGGTTGATCCTGCCAGT-3′) and EukB (5′-TGATCCTTCTGCAGGTTCACCTAC-3′). The newly submitted sequences of *G.* sp1 and *G.* sp2 have been assigned GenBank accession numbers ON141507 and ON141508. The 18S rRNA gene sequences of *G.* sp1 and *G.* sp2 showed a 97.6% sequence identity, with a difference of 38 base pairs.

### Estimating the reaction norm of *Glauconema* phenotypic plasticity

We first measured the distributions of body shapes (length/width ratio) of the trophont and the tomite life stages. Fifty cells were first cultured in 150 × 25 mm Petri dishes with 100 ml food bacteria suspension in autoclaved seawater (OD_600_ ~ 0.3; 25°C), and measured on a compound microscope (Nikon Eclipse Ni-U) by immediately taking photomicrographs, at 30 h for trophonts and 54 h for tomites after inoculation. Then, one chemostat-like culturing device was also used to estimate the body shapes at gradient densities of food bacteria (OD_600_ = 0.5, 0.3, 0.1, 0.05, 0.03, 0.01), with four chambers and a flow-rate of 1 ml/min (Supplementary Fig. S1: Control). For each replicate, the cell length and width of 45 cells were measured after about 8 h in the chambers.

We also measured the swimming speed of cells in the trophont and the tomite stages of the two species, respectively. Swimming speed was measured at two different time points (30 h for trophonts—when all cells are in the trophont stage, and 54 h for tomites—when many trophonts have transformed into tomites) after inoculation. We first isolated cells in the trophont or the tomite stages, and immediately filmed the swimming trajectories of the cells on a Nikon Eclipse Ni-U microscope at 100× magnification. Every video lasted five seconds and was shot at 25 fps (frames per second) with 1636 pixels wide and 1088 pixels high. The swimming speed of 50 cells in each life stage of each strain was measured using the Fiji image analysis platform and manual calibration [71]. The mp4 videos were firstly imported into the software using the function “FFMPEG,” then their “Type” of “Image” was changed into 8-bit, and the “threshold” was adjusted to light background and black labels. Finally, we used MTrack2 in “Tracking” to calculate the distance (*D*: pixels) of cell swimming and the number of frames (*N*) with the parameter “Minimum Object Size (pixels): 10; Maximum Object Size (pixels): 99 999; Maximum Velocity (pixels): 100; Maximum track length (frames): 1.” Cells that emerged in more than 10 frames were included in the calculation. After scale conversion, 1 pixel is about 0.88 μm and one frame is equal to 0.04 s. The swimming speed (*v*: μm/s) was calculated by (*D* × 0.88)/(*N* × 0.04) = 22*D*/*N* (μm/s).

In order to measure the survival curve of tomites, similar culturing conditions in Petri dishes in three replicates were applied as the above for estimating the reaction norm. Cells were first inoculated and cultured for 54 h, when most cells transformed into tomites. Every 24 h, each culture was then thoroughly mixed with a 10 ml pipette, and 1 ml was sampled from the culture. The 1 ml culture was then mixed by vortexing, from which 100 μl was transferred to a Gridded Sedgewick Rafter for three times and tomites were counted (1 mm^2^; Model 1801-G20; cells were fixed with Bouins’ fluid before each counting).

### Sample collection for monitoring the eukaryotic community dynamics of *Glauconema* habitats

**Figure 1:**
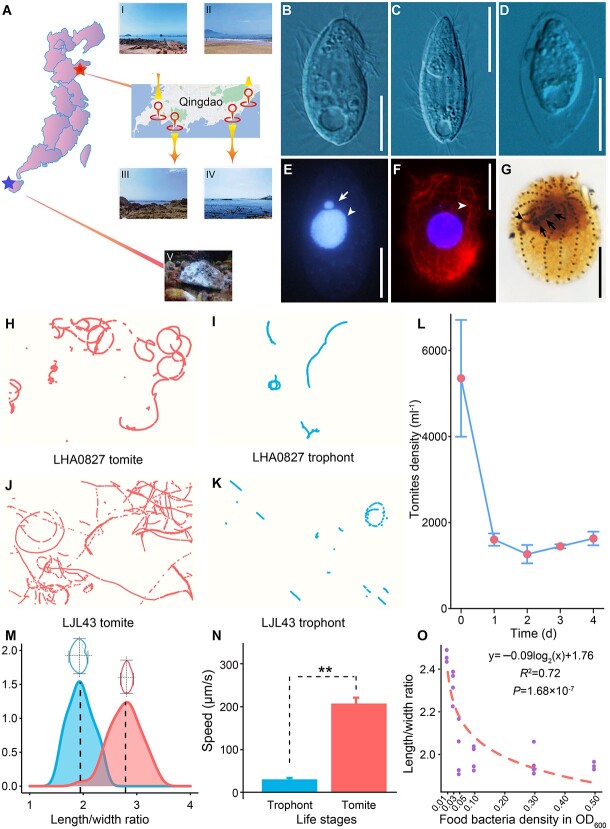
Sampling sites, morphology, and phenotypic plasticity of *Glauconema* spp. (**A**) Sampling sites with successful isolations (*G*. sp1 LHA0827, Qingdao, upper star, IV; *G*. sp2 LJL43, Danzhou, lower star, V) and those for 18S rRNA V8–9 amplicon sequencing to monitor coastal ciliates’ community dynamics (Zhanqiao-I, Huiquan Square-II, Xiaomai Island-III and Sculpture Park-IV. (**B**–**D**) Different life stages of *G.* sp1 LHA0827: trophont (**B**), tomite **(C)**, resting cyst (**D**). (**E**) The macronucleus (arrowhead) and micronucleus (arrow) after Hoechst 33342 staining. (**F**) Arrowhead indicates the cytoskeleton after immune-staining of α-tubulin. (**G**) Paroral membrane (arrowhead) and oral membranelles (arrows). All scale bars are 10 μm. The swimming trajectories of tomites (**H**, **J**) and trophonts (**I**, **K**) were observed for five seconds. (**L**) The survival curve of tomites of *G.* sp1 LHA0827 upon starvation. (**M**) The frequency distributions of the length/width ratio for trophonts (left) and tomites (right). (**N**) The swimming speeds (μm/s) of trophonts and tomites (Mann–Whitney U test, LHA0827, *P* = 1.39 × 10^−15^). (**O**) Fitted curves of the length/width ratios and food-bacteria density.

We isolated *Glauconema* sp1 LHA0827 from sea lettuces in summer coastal waters off Qingdao, northern China. Species identification of isolates was done by live observations on morphology and life cycle, protargol staining, and 18S rRNA gene sequencing (Fig. 1B–G; Table 1; Supplementary Table S1). In order to reveal the seasonal variation of *Glauconema* abundance and guide future collections, we also investigated the biomass of *Glauconema* in coastal waters of Qingdao, by collecting ~10 × 10 cm sea lettuces emerged in seawater every 2 weeks from June to October 2021 with on-site seawater without the algae as control at four closeby spots (Supplementary Table S2), and 18S rRNA V8–9 amplicon sequencing of the phycosphere microflora of sea lettuces sampled (Fig. 1A; Supplementary Fig. S2; Supplementary Table S2; Supplementary Text). We calculated the reads percentage (out of eukaryotes) of *Uronema* spp. (coastal dominant scuticociliates) and *Glauconema* spp. in seawater and sea lettuce samples at each time point to estimate their abundance (Supplementary Fig. S2B). *Uronema* spp. can be seen in all samples, but only one sample for *Glauconema* with extremely low abundance (0.0039%).

**Table 1:** Morphometric features of trophonts and tomites of *Glauconema* sp1 LHA0827 (upper row for each feature) and *Glauconema* sp2 LJL43 (lower row).

**Features**	**Min.**	**Max.**	**Mean**	**SD**	**CV (%)**	**N**
Body length (μm)	26.67	42.51	34.64	3.56	10.28	50
of trophonts	21.25	35.63	29.68	3.08	10.36	50
Body length (μm)	23.63	34.85	29.43	2.65	9.02	50
of tomites	22.84	42.64	31.43	4.11	13.08	50
Body width (μm)	13.04	21.99	17.93	2.24	12.52	50
of trophonts	14.03	28.66	20.59	3.08	14.97	50
Body width (μm)	8.16	14.92	10.67	1.31	12.23	50
of tomites	8.31	16.03	11.20	1.68	14.99	50
Somatic kinety number	12	14	12.63	0.58	4.56	24
of trophonts	12	14	12.68	0.72	5.65	22

Abbreviations: CV, coefficient of variation in %; Max., maximum; Mean, arithmetic mean; Min., minimum; N, number of specimens investigated; SD, standard deviation.

### DNA and RNA extraction for *de novo* genome assembly and annotation of the two *Glauconema* species

For *G.* sp1 LHA0827 or *G.* sp2 LJL43, one single cell was inoculated into 2 ml bacteria suspension of each well on a 6-well plate, and cultured for 30 h. Twenty cells were then transferred to each of 10 150 × 25 mm Petri dishes with 100 ml of food bacteria suspension (OD_600_ ~ 0.3) for 30 h at 25°C. For DNA extraction, cells were first picked using microcapillary pipettes to reduce food bacteria contamination, then harvested by centrifugation at 1500*g* for 5 min, and starved for 2 h to further reduce bacteria contamination. The MasterPure Complete DNA&RNA Purification kit (Cat. No. MC85200; Lucigen, USA) was used to extract the genomic DNA, which was further purified by a Genomic DNA Clean & Concentrator (Cat. No.: ZRC000496; ZYMO).

In order to extract total RNA for genome annotation, we cultured each strain in 400 ml food bacteria suspension (OD_600_ ~ 0.3) in 1 L flasks for 30 (trophonts), 36 (mixture of trophonts and tomites), and 42 h (mostly tomites). For each flask, we transferred and centrifuged 300 ml of upper-layer culture at 1500*g* for 5 min at 4°C. The above MasterPure kit was used to extract total RNA.

### Genome and transcriptome sequencing

For Oxford Nanopore long-read sequencing, the libraries of *G.* sp1 LHA0827 and *G.* sp2 LJL43 were both prepared with the ligation sequencing kit (SQK-LSK109; Nanopore) and were loaded into R9.4.1 flow cells. *G.* sp1 LHA0827 library was sequenced on a MinION sequencing device in the lab and *G.* sp2 LJL43 library on a PromethION platform at NextOmics Biosciences (Wuhan, China). We also performed short-read PE150 sequencing: genomic libraries of *Glauconema* spp. were prepared by the TruSeq Nano kit (Cat. No. 20015964; Illumina, USA) and sequenced by the NovaSeq 6000 System (Illumina) at Berry Genomics, Inc. (Beijing, China).

RNA libraries of the above mass cultures at different life stages for genome annotation were generated using the NEBNext Ultra RNA Library Prep Kit (Cat. No.: E7370L). In order to study possible operon-like structures and alternative splicing, as well as verify the quality of genes annotated, we also performed full-length transcriptome sequencing: PacBio SMRT-bell libraries were prepared by the SMRTbell Express Template Prep Kit 2.0 (Cat. No.: PN 101-853-100), 1–10 kbp size-selected, loaded onto a SMRT cell, and sequenced on a Sequel II system using a 30 h-movie (Pacific Biosciences, CA, USA) at Berry Genomics, Inc. (Beijing, China).

In order to analyze differential gene expression between life stages more accurately than the mass cultures usually with mixed life stages, we also constructed RNAseq libraries using the NEBNext Single Cell/Low Input RNA Library Prep Kit for Illumina (NEB, Cat. No.: E6420S). Briefly, ~20 cells were picked on a dissection microscope from each replicate of three life stages (three replicates for each stage; the initial 20 cells were cultured in 150 × 25 mm Petri dishes with 100 ml food bacteria suspension (OD_600_ ~ 0.3); 30 h for trophonts, 54 h for tomites, and 120 h for resting cysts), and transferred immediately to the cell lysis buffer on ice. NovaSeq 6000 System PE150 (Illumina) sequencing was then applied.

### Macronuclear genome assembly

23.8 and 15.2 Gbp Nanopore and 22.5 and 38.1 Gbp NovaSeq 6000 System raw reads were generated for *G.* sp1 LHA0827 and *G.* sp2 LJL43, respectively. For Nanopore raw reads, we used Guppy v3.3.3 (Oxford Nanopore Technologies) for base calling, and filtered out reads with base quality score < 8 and length < 1000 bp using NanoFilt v2.7.1 [72]. Then, the high-quality reads were mapped to the reference genome of the food bacteria *Pseudoalteromonas* sp. LC2018020214 (NCBI GenBank accession no.: CP066804.1 and CP066805.1), using Minimap2 (-x map-ont) with the default mode [73]. Any reads with a total alignment >80% of their own lengths were considered as contaminated reads. We obtained a total of 20 and 14 Gbp bases for *G*. sp1 LHA0827 and *G*. sp2 LJL43, respectively.

For Illumina sequences, adaptor-trimming and low-quality-reads filtering were performed with fastp v0.20.1 with the following parameter: “-u 20 -q 20”. The resulting reads were mapped to the food bacteria reference genome using BWA v0.7.17 with default settings [74]. We extracted unmapped data using SAMtools v0.1.9 [75] with -bf 12 and obtained 12 and 20 Gbp Illumina PE150 bases for *G.* sp1 LHA0827 and *G.* sp2 LJL43, respectively.

For *G*. sp1 LHA0827, we first estimated the macronuclear genome size with high-quality Nanopore reads using wtdbg2 v2.5 [76] and Flye v2.8 [77] (see detail in Supplementary Text). The draft genomes assembled by Canu v2.1.1 with the parameter: genomeSize = 200 m [78] received three rounds of polishing with Racon v1.4.3 with the default parameters [79] and another three additional rounds with Pilon v1.24 with the parameter: “--fix snps,indels” [80]. To further exclude bacterial contamination, contigs with GC content higher than 27% were removed for *G*. sp1 LHA0827, based on GC-content-distribution peaks of contigs (Supplementary Fig. S3A). Contigs were also queried against the bacterial genome database on 19 June 2021, downloaded from NCBI (ftp://ftp.ncbi.nlm.nih.gov/genomes/refseq/bacteria/), using BLASTN v2.10.1 (e-value: 1e-5) [81]. Any contigs with ≥80% identity and cumulative hit length ≥60% were filtered out. We also removed the contigs with sequencing coverage <20×. Finally, we also checked for and then kept the contigs that were filtered out as false negatives and found one contig containing the SSU-rRNA gene, as well as another two contigs with one telomere for *G*. sp1 LHA0827. Assemblies were evaluated using QUAST v5.0.2 with default settings [82] and BUSCO v5.2.2 [83] with the settings -l alveolata_odb10 --augustus --augustus_species tetrahymena. Abundant (C_4_A_2_)_n_ repeats are prevalent in the contig ends, which are also used in the closely-related *Tetrahymena* specie, a minimum of two copies of C_4_A_2_ were required to designate a contig telomere. Assembling and filtering details for *G*. sp2 LJL43 are in Supplementary Fig. S3B. The inference of stop codon usage was used by codetta v2.0 [84].

### Gene prediction and annotation

The repeated regions in the genome were first detected using RepeatMasker v4.1.0 with “-e rmblast” [85] and RepeatModeler v2.0.1 with “-LTRStruct” [86]. tRNAscan-SE v2.0.9 with default settings [87] was used to identify the transfer RNAs (tRNAs). Then ribosomal RNA (rRNA) genes in the genome were also parsed out by RNAmmer v0.1.2 [88] with the settings -S euk.

To identify protein-coding genes, first, we used fastp to trim the RNASeq data of different life stages mass cultured with the default parameters and mapped the trimmed data to the reference genome of the food bacteria with Hisat2 v2.1.0 [89], and then removed the contaminated reads using Samtools v0.1.9. *De novo* gene prediction and transcriptome-based methods were both applied. Trinity v2.21.0 [90] with the parameter: “--seqType fq” was used for *de novo* transcripts assembling and reference-guided transcripts assembling. The Hisat2 mapping results were converted to the bam format, which was used to predict the gene structures by Braker2 v2.1.6 [91] and StringTie v1.3.7 with default settings [92]. The combination of the above *de novo* and reference-guided assembled transcripts was used as the cDNA evidence for Augustus v3.3.3 [93] to train the gene prediction model. All of the gene sets from GlimmerHMM with default parameters, GenomeThreader with “-translationtable 6,” the Analysis and Annotation Tool (AAT) Package with “--dds ‘-f 100 -i 20 -o 75 -p 70 -a 2000’ --filter ‘-c 10’ --gap2 ‘-x 1’,” PASA with “-C -R --ALIGNER gmap,” and Augustus with the only stop codon TGA were merged to produce the eventual gene sets using EVidenceModeler v1.1.1 with “--segmentSize 100000 --overlapSize 10000” and “--stop_codons TGA --min_intron_length 10” [94]. The non-redundant protein database (NR) was used to annotate protein-coding genes by BLASTP (e-value 1e-5 -word_size 3 -num_alignments 20 -max_hsps 20 -show_gis). The gene name was derived from the best hit. The GO annotation was done by OmicsBox v1.4.11, and the KEGG pathway annotation was merged by the results of KAAS (KAAS—KEGG Automatic Annotation Server: https://www.genome.jp/kegg/kaas/; BBH method), KofamScan v2022-06-02, and eggNOG-mapper v2 [95–98]. Then, the KOs (KEGG Orthology) were converted to the Reference hierarchy (ko, reference pathway highlighting KOs) by KEGG Mapper (https://www.genome.jp/kegg/mapper/search.html) [99].

The MITOS and GeSeq were used to annotate the mitogenomes with genetic code 4 of “the mold, protozoan, and coelenterate mitochondrial code and the mycoplasma/spiroplasma code.” The protein-coding genes were further verified by the NCBI Open Reading Frame Finder (https://www.ncbi.nlm.nih.gov/orffinder, accessed on 2 May 2022) and annotated by searching against the NCBI non-redundant protein sequences (NR) database with BLASTP. The ribosomal RNA (rRNA) genes were searched against the rRNA genes of *Uronema marinum* (NCBI Gene IDs: 37625978 and 37625943) by BLASTN. The tRNAscan-SE v2.0 was used to predict the tRNA genes with the default mode. The central repeat regions were found by TRF v4.09 [100].

### Comparative genomics and gene family analysis

We used BLASTP to find similar proteins of the two *Glauconema* macronuclear genomes with an E-value threshold of 1e-05, then we used the function “File Merge For MCScanX” in TBtools to convert the gff3 to a concise format. Finally, the results of the collinearity between genomes were generated by Quick Run MCScanX Wrapper in the TBtools v1.106 [101]. The visualization of collinearity is done using “Advanced Circos” in TBtools [102]. Collinearity analysis within the genome also uses the above approach.

To identify gene families, we downloaded seven high-quality macronuclear genome sequences of ciliates—*Halteria grandinella* (GCA_006369765.1), *Ichthyophthirius multifiliis* (GCF_000220395.1), *Oxytricha trifallax* (GCA_000295675.1), *Paramecium tetraurelia* (GCA_000165425.1), *Pseudocohnilembus persalinus* (GCA_001447515.1), *Tetrahymena thermophila* (GCA_000189635.1) from NCBI, and *Euplotes vannus* (Mar 2018) from Ciliates Genome Database (http://ciliates.org/). We selected protein sequences derived from the longest transcripts for every gene by CD-hit, respectively, and discarded those with fewer than 50 amino acids [103]. Finally, we got 212 756 proteins, which were used as the input of OrthoFinder [104] to infer the phylogenetic orthology with the parameters: -f /input_folder -M msa -T fasttree -T 28 -a 28 -S diamond. We obtained three time points as primary calibrations from the Timetree database (http://www.timetree.org/; *E. vannus* vs. *Paramecium tetraurelia*; *Tetrahymena thermophila* vs. *Paramecium tetraurelia*; *Ichthyophthirius multifiliis* vs. *Tetrahymena thermophila*). Then, r8s [105] was used to build the ultrametric tree based on the estimates provided by Timetree, and the expansion and contraction analyses of gene families were performed using CAFE v4.2.1 [106]. iTOL v6 was then used to display and annotate the tree [107].

### RNAseq-based gene expression in different life stages

After sequencing, we obtained an average of 10.37, 9.96, and 4.07 million clean reads for each sample of trophonts, tomites, and resting cysts, respectively. For *Glauconema* sp1 LHA0827, RNAseq clean reads from low-input RNA library constructions of different life stages were mapped to the genome of *G.* sp1 LHA0827 using Hisat2 v2.1.0, then sam files were converted to bam format using samtools v1.3.1. StringTie v2.1.5 with the setting -e -B -G and prepDE.py3 script in StringTie were used to calculate the expression level of each gene. Genes with significantly different expression levels were identified by DESeq2 v1.32.0 [108] with the setting |log_2_(Fold change)| ≥ 1 and *P*_adj_ < .05. OmicsBox v1.4.11 was used for the Gene Ontology (GO) analysis. GO and KEGG pathway enrichment analyses of the significantly differential expressed genes were done by clusterProfiler v4.0.2 (*P*_adj_ < .05 and *q-*value < .05) [109]. 13 DEGs (1.75% of all) between trophonts and tomites, and 19 for trophonts vs. resting cysts (2.66% of all), belong to the expanding gene families (Supplementary Tables S3 and S4).

In order to calculate dN/dS of the genes, we first retrieved the homologous genes of *G.* sp1 LHA0827 and *G.* sp2 LJL43. ParaAT v2.0 was used to align the protein sequences of genes and match the aligned protein sequences with the corresponding DNA sequences with parameters: -m clustalw2 -f axt -g [110, 111]. For genes with dS < .75, the dN/dS was calculated by KaKs_Calculator v2.0 with -m YN [112, 113], and the Jukes-Cantor Model was applied to account for multiple substitutions [114].

### qPCR

The culturing and RNA extraction procedures were consistent with those for gene expressions in different life stages. The Hieff NGS Single Cell/Low Input cDNA Synthesis & Amplification Module kit (Cat. No.: 12500ES24; Yeasen Biotechnology, Shanghai) was used for cDNA generation, with oligo-dT primers to target eukaryotic mRNA. The cDNA was used as the template for qPCR. Then qPCR was done with the protocol of Hieff UNICON Universal Blue qPCR SYBR Green Master Mix (Cat. No.: 11184ES08; Yeasen Biotechnology, Shanghai) with 20 μl total reaction system with three replicates per life stage per gene. The primers of the two target genes for the qPCR were shown in Supplementary Table S5. The expression differences of the same gene in different life stages were calculated by comparing with the control gene *jmjC*, which has stable expression levels across different life stages based on RNAseq analysis, and its homolog *jmj1* (absent in *Glauconema* genomes) is frequently used as a qPCR control gene in *Tetrahymena* [115], using the 2^-ΔΔCt^ method.

We also analyzed the relative copy number of each gene at the DNA level in different life stages, using the RoomTemp Sample Lysis Kit (Cat. No.: P073; Vazyme, China) to lyse cells, which were directly used as the qPCR templates (three cells per replicate). Mapping and significance analyses were implemented by GraphPad Prims v9.0.0 (www.graphpad.com).

### RNAi experiments

The PCR products of the two target genes (*pgk1*: tig082700000136.101; RNA binding protein: tig082700000279.64), of which primers were shown in Supplementary Table S5, were ligated into a L4440 plasmid. The L4440 plasmid was extracted by Plasmid Mini Kit II (Cat. No.: D6945-01; Omega Bio-Tek, USA). Then, the L4440 plasmid was linearized by using reverse amplification primers (Supplementary Table S5) at two restriction sites (HindIII and Xbal). The PCR products of the target genes and the linearized L4440 plasmid were reconstituted using ClonExpress II One Step Cloning Kit (Cat. No.: C112; Vazyme Biotech Co., Ltd, Nanjing). After that, the L4440 plasmid linked with the target fragment was transformed into *Escherichia coli* HT115-competent cells, which were then plated on a LB medium plate containing tetracycline (12.5 μg/ml) and ampicillin (50 μg/ml). Positive clones were cultured and induced by 0.4 mmol/L IPTG to express dsRNA as the treatment. The bacteria without IPTG induction were cultured simultaneously as the control. Afterwards, bacteria in the control and the treatment were rinsed twice with sterile seawater to remove medium and IPTG. After the *E. coil* cells were digested, dsRNA of the target gene expressed by the L4440 plasmid was discharged into the cytoplasm of hosts to mediate the target mRNA degradation. To ensure that population density did not affect body shape, we used a chemostat-like system to keep relatively stable food bacteria densities (OD_600_ = 0.1) (IPTG or non-IPTG groups), with four chambers for each group and a flow-rate of 212 μl/min for 84 h (Supplementary Fig. S1). For each replicate, the cell length and width of 45 cells was measured after ~48 and 60 h and RT-qPCR was used to measure the relative expression of the target gene.

## Results

### Reaction norm of body shape follows a power-law distribution

We observed typical *Glauconema* phenotypic plasticity: when food bacteria were abundant, cells were broad-bean-shaped and slowly-moving trophonts, but transformed into fusiform-shaped and fast-swimming tomites when food bacteria were scarce, with a much narrower body shape than that of the trophonts (length/width ratios: 2.78 vs. 1.95, *t*-test, *P* = 9.63 × 10^−29^) and much faster swimming speed (207.50 vs. 30.86 μm/s; *P* = 6.94 × 10^−16^) (Fig. 1M and N; Supplementary Table S6). The swimming trajectories also reflect the larger range of activity of tomites (Fig. 1H and I). Trophonts also occasionally form resting cysts (Fig. 1D).

To quantify phenotypic plasticity, we explored the reaction norm of body shape (BS) vs. food-bacteria density (FBD) at 25°C, using a chemostat-like culturing system to maintain bacterial density at relatively stable levels (Supplementary Fig. S1). The body shape was estimated by the mean length/width, and food-bacteria density was approximated by OD_600_ measurement (Supplementary Table S7). These results revealed a power-law reaction norm: *BS* = −0.09log_2_(*FBD*) + 1.76, *R*^2^ = 0.72, *P* = 1.68 × 10^−7^ (Fig. 1O), indicating that the higher the food-bacteria density, the wider the cells (more trophonts), consistent with the life cycle from microscopic observations. Analyses of the length/width ratios from each experiment revealed widespread bimodal distributions, especially at lower bacterial density (Supplementary Fig. S4). The relative heights of the two peaks changed with food density, with relatively more tomites appearing at lower food densities. This infers that the life stages might be controlled by a single switch gene.

### *De novo* assembled macronuclear genome demonstrates high gene number

**Figure 2:**
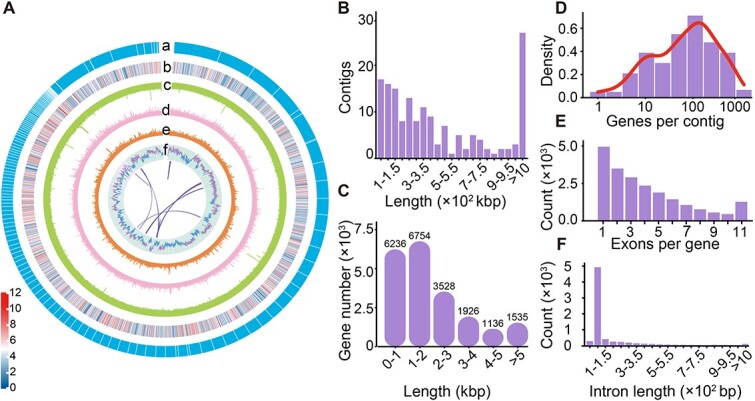
Genomic features of *Glauconema* sp1 LHA0827. (**A**) Characteristics of the assembled contigs of *G.* sp1 LHA0827. a–f represent the contigs, the distribution of gene density (corresponding to the color legend on the up-left corner), depth of coverage of Nanopore sequences, depth of coverage of Illumina sequences, GC density and GC skew calculated in 20 kbp sliding windows (5 kbp step size). The interconnecting lines represent collinear genes within the genome. (**B**, **C**) The distributions of contig length and gene length with introns. (**D**–**F**) The distributions of gene number per contig, exons per gene, and the intron length.

Genomic resources for *Glauconema* were previously absent, imposing a barrier to understanding the molecular basis of phenotypic plasticity. Thus, using Nanopore long reads and Illumina PE150 short reads, we assembled a high-quality macronuclear genome of *Glauconema* sp1 LHA0827 with a size of 91.27 Mbp, containing 159 contigs without gaps, with N50 1.19 Mbp and the longest contig being 4.29 Mbp (Fig. 2A and B; Table 2; Supplementary Table S8) (see assembling details in Materials and Methods, Supplementary Fig. S3). The number of contigs with at least one telomere (telomere sequence repeats ([C_4_A_2_]_n_)) in the *G*. sp1 LHA0827 genome is 89% of the total contig number (39% for those with two telomeres) (Table 2; Supplementary Table S8).

**Table 2:** The macronuclear genomic features of the two *Glauconema* species.

**Species**	***G*. sp1 LHA0827**	***G*. sp2 LJL43**
Genome size (bp)	91 273 816	98 872 637
Contigs number	159	181
N50 (bp)	1 185 523	1 461 257
N70 (bp)	571 647	586 454
Longest contig (bp)	4 293 306	4 082 163
Mean G/C content	25%	23%
Gaps	0	0
2 telomeres	62	122
1 telomere	80	51
Number of contigs with 18S rRNA genes	1	14
G/C content of contigs with 18S rRNA genes	39%	38%
Gene number	21 115	28 909
Gene length (bp)	2152	2275
rRNA number	35	164
tRNA number	445	503
Gene BUSCO	86%	94%

Using transcriptome-based, homology-based, and *de novo* gene-prediction pipelines, we annotated 21 115 genes for the macronuclear genome, with a BUSCO score with a genome model of 88% and 25% G/C content (Fig. 2A; Table 2). The gene number, G/C content, and other genomic features are comparable to those for other ciliate genomes in the same *Oligohymenophorea* class, while greatly differ from the nanochromosomal genomes with low N50 in the *Spirotrichea* class (Table 3). The mean gene length is 2.15 kbp (Fig. 2C and D; not including UTRs). On average, each gene contains ~4 exons, with a median size of 240 bp (Fig. 2E), and ~3 introns with a median size of 69 bp (Fig. 2F). We also identified 35 rRNA and 445 tRNA genes (Table 2). As in *Tetrahymena thermophila*, TGA is the only stop codon, while TAA and TAG are both reassigned to encode glutamine (Supplementary Table S9).

**Table 3:** The details of macronuclear genomes of ciliates in the comparative genomic analyses.

**Species**	**G**	**N**	**N50**	**L**	**GC**%	**Gene No.**	**D**
*Euplotes vannus*	84.8	37 486	2.7	0.04	37	43 338	511
*Glauconema* sp1 LHA0827	91.3	159	1186	4.30	25	21 115	231
*Glauconema* sp2 LJL43	98.9	181	1461	4.10	23	28 909	292
*Halteria grandinella*	64.0	40 422	2.1	0.07	43	17 815	278
*Ichthyophthirius multifiliis*	47.8	1375	66	0.40	16	8062	169
*Oxytricha trifallax*	67.2	22 363	3.7	0.06	31	24 578	366
*Paramecium tetraurelia*	72.1	697	413	1.00	28	39 642	548
*Pseudocohnilembus persalinus*	55.5	288	368	2.00	19	13 186	238
*Tetrahymena thermophila*	103.0	1148	521	2.20	22	26 460	256

G, Genome size (Mbp); N, Contigs number; N50 in kbp; L, Longest contig (Mbp); GC%, mean G/C content; Gene No., Gene number; D, Gene density (genes/Mbp). *E. vannus*, *H. grandinella*, and *Oxytricha trifallax* belong to the class *Spirotrichea*; *Glauconema* sp1 LHA0827, *Glauconema* sp2 LJL43, *Ichthyophthirius multifiliis*, *Paramecium tetraurelia*, *Pseudocohnilembus persalinus* and *Tetrahymena thermophila* all belong to the class *Oligohymenophorea*. Accession numbers are shown in the Materials and methods section.

The linear mitochondrial genome is 53 226 bp in size with 40 protein-coding genes, 6 tRNA and 3 rRNA genes (Supplementary Fig. S5A; Supplementary Table S10). It contains central repeat regions, but lacks telomeric repeats, in contrast to the macronuclear genome (Supplementary Tables S8 and S10). The mitochondrial codon usage is also different from that of the macronuclear genome, with TGA encoding tryptophan and TAA/TAG being stop codons (Supplementary Table S9).

### Low variation of *Glauconema* phenotypic plasticity inferred from comparison between congeners

To explore the variation of phenotypic plasticity in *Glauconema*, we isolated another species, *Glauconema* sp2 LJL43, from coastal waters of Hainan Province, southern China, and compared its phenotypic plasticity with that of *G*. sp1 LHA0827. *G*. sp2 LJL43 has a similar life cycle and swimming patterns to *G*. sp1 LHA0827 (Fig. 1J and K; Table 2; Supplementary Text; Supplementary Fig. S6; Supplementary Tables S1, S6, S8, S9, and S11). Whereas there is a significant difference in the body shape of trophonts between *G*. sp2 LJL43 and *G*. sp1 LHA0827 (*t*-test, *P* = 3.51 × 10^−16^), there is no difference for tomites. The same tomite body shape and swimming speed in two species suggest that they are experiencing similar selective regimes for this particular body shape and swimming, or that the limit of phenotypic plasticity has been reached, meaning that tomites cannot become narrower or swim faster, although data from more species are needed to reach a conclusion.

**Figure 3:**
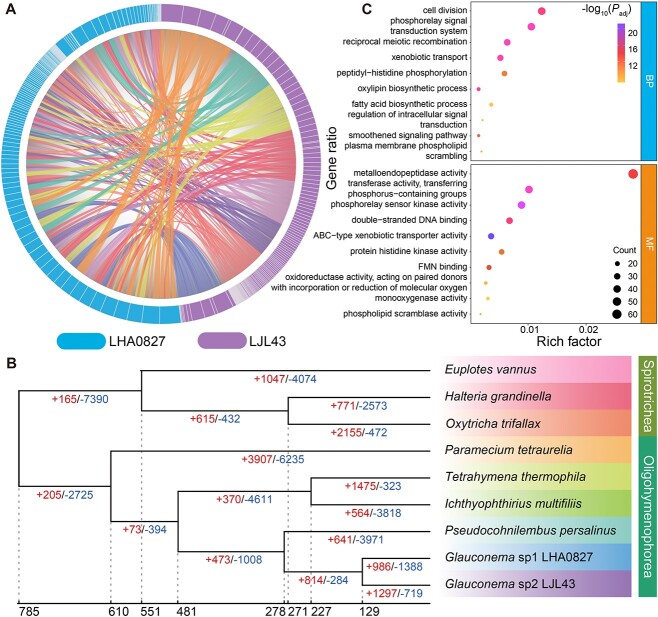
Comparative genomics and evolution of *Glauconema* spp. macronuclear genomes. (**A**) The co-linearity of two *Glauconema* macronuclear genomes. (**B**) The gene-family comparisons. Dynamic evolution of gene families among nine ciliates (the all-to-all blast to determine the similarities between genes). The numbers at the bottom represent the inferred time in million years (based on correlated rates clock). The left and right numbers under the branches represent the expanded or contracted gene families in each linage, respectively. Note that due to the high similarity of the genomes of the two *Glauconema* species, numerous unique gene families shared between them lead to the inference of many contracted gene families in other species. (**C**) The GO enrichment of genes in expanding gene families of *Glauconema* (Supplementary Table S13). BP and MF represent Biological Process and Molecular Function, respectively.

To further investigate the evolution of *Glauconema* spp., we performed *de novo* assembly and annotation on *G*. sp2 LJL43 and obtained its macronuclear genome with a BUSCO score of 88%, totaling 98.87 Mbp in size. The mitochondrial genome encodes 43 complete protein-coding genes (Supplementary Fig. S5B; Supplementary Table S12). We then compared the macronuclear genomes of *G*. sp1 LHA0827 and *G*. sp2 LJL43 by first performing collinearity analyses (Figs 2A and 3A; Supplementary Fig. S6H). Few collinear blocks were observed within each genome (Fig. 2A; Supplementary Fig. S6H). However, 8964 gene pairs were detected with collinearity between the two genomes (Fig. 3A), representing 43% and 31% of the total number of genes for *G*. sp1 LHA0827 and *G*. sp2 LJL43, respectively, which is equivalent to 22% and 25% of their genome sizes, demonstrating the high divergence between the two species.

We also conducted gene-family analysis on the macronuclear genomes of *G*. sp1 LHA0827, *G*. sp2 LJL43, and seven other ciliates. In total, we identified 29 398 gene families. The high similarity in gene families between the two *Glauconema* genomes was consistent with their almost identical phenotypes and life histories (Fig. 3B). As expected, the ultrametric tree, calibrated with the time points from the Timetree database, indicated that they are the closest to each other among the nine ciliates, and the divergence of the two *Glauconema* species from the common ancestor occurred ~130 million years ago (Fig. 3B).

A gene family comprises multiple paralogs originating from the duplication of a single ancestral gene, typically sharing similar functions. Over evolutionary time, members within the family can increase (expansion) or decrease (contraction), which could be associated with natural selection, with beneficial gene families expanding under positive selection, and contracting gene families reflecting loss by non-functionalization and genetic drift [116–119]. Based on the gene families of the nine ciliates studied, we infer that the common ancestor of the two *Glauconema* species underwent expansions of 814 gene families (including 32 DEGs, Supplementary Tables S3 and S4) and contractions of 284 gene families. The expanded genes are involved in rapid cellular responses to extracellular signals, resistance to intracellular toxic substances, lifespan, cell cycle, and so on (Fig. 3C; Supplementary Table S13).

### Phenotypic plasticity is under strong purifying selection

**Figure 4:**
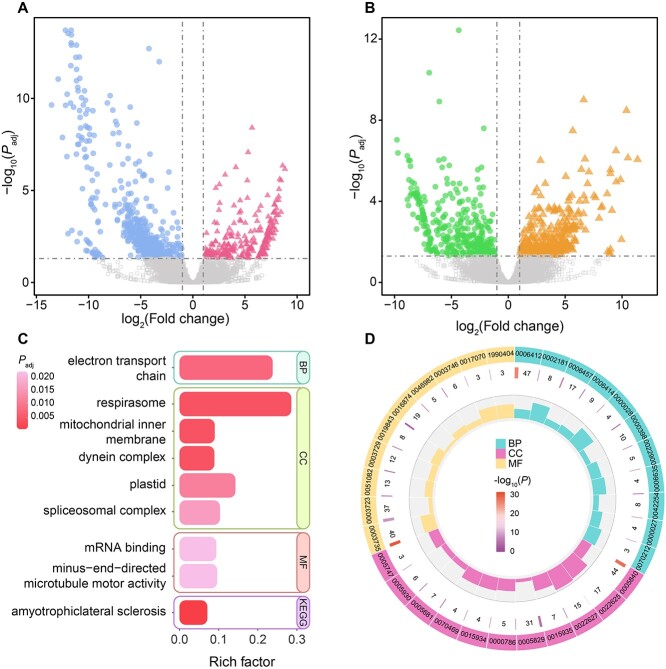
Differential expression and enrichment analyses. (**A**, **B**) The differential gene expression between tomites vs. trophonts (A) and resting cysts vs. trophonts (B) of *Glauconema* sp1 LHA0827. When compared with trophonts, circles represent genes that are significantly down-regulated, and triangles represent genes that are significantly up-regulated in tomites/resting cysts (A/B). Significant difference: |log_2_(Fold change)| ≥ 1 & *P*_adj_ < .05. (**C**) The GO enrichment and KEGG (KG) pathway enrichment analyses of significantly down-regulated genes in resting cysts, (vs. trophonts). BP, CC, and MF represent Biological Process, Cellular Component and Molecular Function, respectively. (**D**) The GO enrichment of down-regulated genes in tomites, when compared with trophonts (Supplementary Table S14). The outermost blocks represent the GO IDs. The numbers in the middle circle indicate the gene counts enriched for the corresponding GO ID, with different colors denoting distinct *P* (color legend shown at the center). The innermost circle illustrates the proportion of significantly differentially-expressed genes enriched relative to the total number of genes associated with the GO ID.

Certain genes show differential expression at different life stages, indicating a possible association with the molecular mechanisms underlying phenotypic plasticity. Gene-enrichment analyses on these differentially expressed genes (DEGs; 246 significantly up-regulated genes and 498 down-regulated genes in tomites vs. trophonts) in *G.* sp1 LHA0827 indicate that many necessary biological processes are down-regulated in tomites, including protein translation and modification, ribosome assembly, electron transport chain, mRNA splicing, and fatty acid beta-oxidation (Fig. 4A; Supplementary Table S3). Consistently, the genes associated with many core cellular components, such as ribosomes, nucleosomes, respirasome, and axoneme for cilia movement, also have decreased expression (Fig. 4D; Supplementary Table S14). In contrast, autophagy is significantly up-regulated in tomites. We speculate that such up-regulation cuts down energy for major life activities and reallocates it for temporary food searching (Supplementary Table S3). This is highly consistent with the survival curve of tomites starting to drop 24 h after the initial appearance of starvation (Fig. 1L). Thus, tomites are in a near-death state.

Based on our observations and previous literature, resting cysts are generally transformed directly from trophonts [61]. We thus analyzed the differential gene expression between resting cysts and trophonts and identified 344 significantly up-regulated and 369 down-regulated genes in resting cysts compared with trophonts (Figs 1D and 4B; Supplementary Table S4). There were only a few biological functions enriched in significantly down-regulated genes (Fig. 4C; Supplementary Table S15). Translation and respiration are among the most down-regulated molecular functions, with decreased expression in mRNA binding, electron transport chain, and respiratory energy supply.

**Figure 5:**
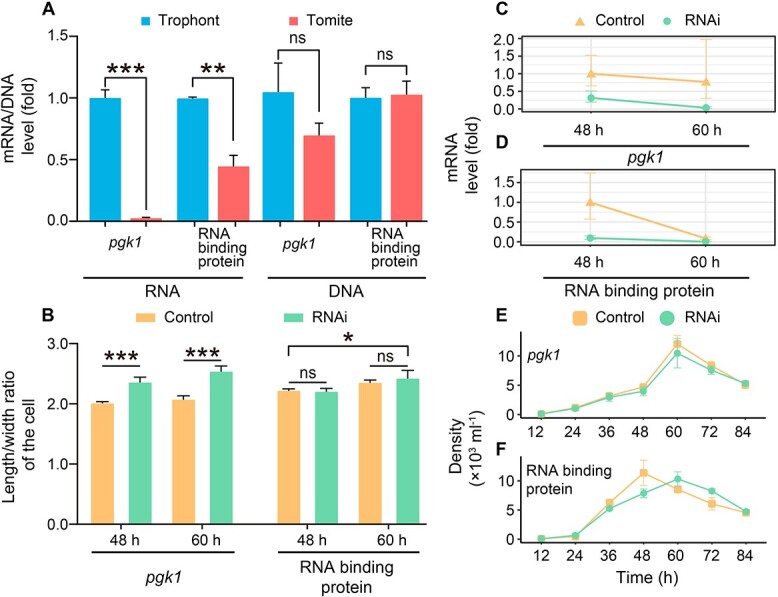
qPCR and RNAi results of *Glauconema* sp1 LHA0827. (**A**) The relative expression levels of trophonts vs. tomites and the relative chromosomal copy number for two genes (*pgk1* and RNA binding protein). ^*^^*^^*^P < .001, ^*^^*^P < .01, ^ns^*P* > .05, based on *t*-tests. (**B**) The mean length/width ratios of the cells at different time points under knockdown (RNAi) and Control conditions with constant food density (OD_600_ = 0.1). (**C**, **D**) The relative expression levels of trophonts vs. tomites for *pgk1* and RNA binding protein at different time points under knockdown (RNAi) and Control conditions. (**E**, **F**) The cell densities over time of the two groups (RNAi or Control) for the two target genes (E: *pgk1*; F: RNA binding protein) in the chemostat-like culturing system.

To ensure the reliability of our differential gene-expression analysis, we performed RT-qPCR and RNAi to support the expression patterns and investigate phenotypic plasticity effects of two genes (tig082700000136.101, Phosphoglycerate Kinase 1, *pgk1*, involved in glycolysis; tig082700000279.64, RNA binding protein; Supplementary Table S3), which were drawn from the top eight down-regulated genes in tomites. The expression patterns of the RT-qPCR supported those from the low-input RNAseq-based differential expression analysis (Fig. 5A; Supplementary Tables S3 and S16). Consistently, RNAi experiments also demonstrated that the body shape changed in the expected direction with the RNAi-induced changes in expression of these two genes (Fig. 5B–F; Supplementary Tables S5, S16, and S17). These findings thus supported the association of phenotypic plasticity with multiple differentially expressed genes.

Previous studies have suggested that ciliates under stress may temporarily increase the copy number of specific chromosomes, in which some possibly adaptive regulatory genes reside [120]. For *pgk1* and the RNA-binding protein, we analyzed their copy number changes at the DNA level of trophonts vs. tomites of *Glauconema* sp1 LHA0827, using qPCR at each life stage. We find that neither gene has a significant difference at the DNA level in the two life stages (Fig. 5A). This suggests that the phenotypic plasticity of *Glauconema* is not regulated by copy number changes at the DNA level, which is a more energy-consuming task, but by directly altering gene-expression levels. This is highly consistent with that *pgk* expression at the mRNA level is under post-transcriptional regulation in the parasitic protozoa *Trypanosoma brucei* [121].

In order to explore the evolutionary forces on the DEGs associated with phenotypic plasticity, we calculated the ratios of the number of non-synonymous substitutions per non-synonymous site to the number of synonymous substitutions per synonymous site (dN/dS) for the homologous DEGs in the two *Glauconema* genomes. The Jukes-Cantor model was applied to account for multiple substitutions, and only genes with dS < 0.75 were considered due to the long evolutionary history. dN/dS values of almost all DEGs are much <1 (median 0.04), and not significantly different from those of non-DEGs (*P* > .05; Supplementary Table S18), indicative of strong purifying selection, although the possibility that other selective forces exist in genes not passing the filters cannot be excluded. As expected, phenotypic plasticity is under functional constraint and preserved over long-term evolution.

## Discussion

We explored the regulation and evolution of phenotypic plasticity, by deriving the reaction norm, *de novo* assembling and annotating macronuclear genomes, analyzing and verifying differential gene expression between life stages, and conducting comparative genomic analyses of the marine ciliates *Glauconema* spp. We discovered a power-law reaction norm of body shape vs. density of the food bacteria. However, the extent to which feeding preference for different bacteria or other physicochemical factors (e.g. pH and temperature) alters the phenotypic plasticity remains an open question. Measuring the phenotypic responses to more environments is thus needed for a thorough characterization of phenotypic plasticity, necessary for testing whether phenotypic plasticity is adaptive.

Our study revealed hundreds of differentially expressed genes associated with trophont-tomite transformation, and verified two of them with RT-qPCR and RNAi, along with canonical genes, such as mTOR (a crucial kinase regulating cell growth; [122, 123]), stably but not differentially expressed in any life stage, collectively showing that the phenotypic responses involve actions of numerous genes at the cellular level. However, the causal relationship between phenotypic plasticity and changes in gene expression, or whether the change in expression precedes the phenotypic alteration or occurs as a consequence thereof, needs further exploration. Also, whether more genes are involved in the regulation of phenotypic plasticity under a wider range of culturing conditions remains an open question. Therefore, it is too early to conclude that phenotypic plasticity relies solely on expression changes in the genes we tested. Also, we have yet to identify the switch gene in *Glauconema* that triggers the phenotypic plasticity response to starvation, as well as the role of epigenetic factors. The evolutionary processes acting on key genes also remain unclear, and additional population genomic data would be valuable, especially for a quantitative genetics analysis of this phenomenon [124–126]. However, due to the extremely low abundance of *Glauconema* in natural seawater (Supplementary Fig. S2B), we were only able to obtain two isolates for analysis. Despite these, the highly similar reaction norms and life history features, as well as the predominantly dN/dS values <1 of DEGs between life stages, support that the phenotypic plasticity response to food bacteria density is likely to be a conserved trait, which could be maintained by natural selection over the long evolutionary history.

Some genes show up-regulation upon encystment (Fig. 1B and D; cells shrink during this process), with enrichment in one particular pathway associated with human muscle cell atrophy (amyotrophic lateral sclerosis, ko05014). They function as axonemal proteins and/or energy or plasma-membrane choline transporters in resting cysts and also have homologues in most model ciliates (Supplementary Table S19). Such findings might provide a novel research model for studying the molecular genetics associated with cell-shrinkage.

We verified two genes’ function in phenotypic plasticity (*pgk1*, and the RNA binding protein tig082700000279.64), out of hundreds of differentially-expressed genes. *pgk1* is ubiquitously present across eukaryotes. Many studies have demonstrated that increased *pgk1* expression is conducive to energy synthesis and autophagy processes (genes related to autophagy, such as *atg6*, are known to exist in ciliate genomes) [127–130]. Additionally, *pgk1* has been shown to interact with genes associated with resistance to external environments, such as Hsp90 protein, which plays a critical role in tolerance to high temperatures in *Tetrahymena* [59, 131]. Thus, we hypothesize that lower energy synthesis in the tomite stage of *Glauconema* results from decreased *pgk1* expression, which is induced by food bacteria shortage. Such process could be done so by *pgk1* regulating the ratio of ATP and AMP [132, 133]. Although more tests on the direct causal effects of these genes are needed, our RNAi results support this hypothesis (Fig. 5B and C). ATP depletion has been reported to affect the expression of multiple genes and to alter transcriptional regulation in different cellular contexts. Knocking down the RNA-binding protein (tig082700000279.64) also reduces cell metabolism (Fig. 5B and D). Although the function of the RNA-binding protein has not been specified, a homologous protein has been annotated in *Tetrahymena* [134].

**Figure 6:**
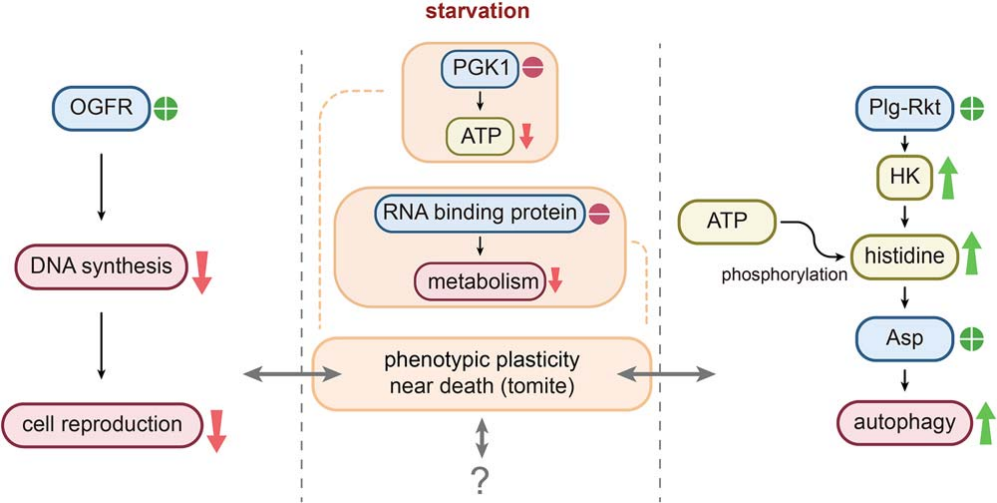
A schematic model of phenotypic plasticity regulation in *Glauconema*. The plus sign in the circle indicates genes that are significantly up-regulated in tomites compared with trophonts, while the minus sign in the circle indicates genes that are significantly down-regulated. A down arrow signifies a decrease in the substance or process, whereas an up arrow indicates an increase.

We identified additional differentially expressed genes possibly associated with phenotypic plasticity. Whereas the prevalence of these genes in ciliates is notable, their precise functions within *Glauconema* have yet to be investigated. As a result, understanding their potential impact on the life history of *Glauconema* can now only be inferred through extrapolation from their established functions in distantly related taxa. Among these genes, we found OGFr (opioid growth factor receptor; tig082700000016.335), a transmembrane protein expressed in various tissues, including the nervous and immune systems of mammals. Although ciliates lack an immune system per se, they possess a hormonal system that may function partly like the immune system [135]. Previous studies have shown that up-regulation of OGFr expression affects DNA synthesis [136], and we observed a significant increase in its expression in tomites compared to trophonts (Supplementary Table S3), suggesting that it may play a role in inhibiting cell division and reproduction of tomites (Fig. 6; cell division of tomites was never observed or reported). Another transmembrane protein identified in our study is PLG-R_KT_ (plasminogen receptor (KT); tig082700000116.6), which may respond to external signals and regulate cellular responses to starvation [137]. Up-regulation of PLG-R_KT_ may activate the signal transduction process of histidine kinase (HKs), leading to phosphorylation of histidine and subsequent phosphotransfer to an aspartate residue of relevant response regulators [138, 139]. Phosphorylated aspartate has been shown to enhance autophagy, which is consistent with our findings of significantly up-regulated expression of genes associated with autophagy in tomites (Supplementary Table S3) [140]. We speculate that such up-regulation cuts down energy for major life activities and reallocates it for temporary food searching. Moreover, tomites transform into trophonts in the presence of sufficient food bacteria, at which point the autophagy pathway in *Glauconema* may be curtailed. Based on the above, we propose a tentative schematic model for the genetic regulation of phenotypic plasticity in this marine unicellular eukaryote, while acknowledging the existence of unresolved factors and unexplored variables (Fig. 6).

We speculate that tomites are in a state of decline and are specifically for searching food, as they tend to vanish soon after emerging (Fig. 1L), especially in the two species studied here, which do not typically form resting cysts in lab cultures. Binary fission of tomites was observed neither in our initial study on *Glauconema* [60], nor in any previous reports, so they represent a terminal state analogous to resting cysts. Tomites exhibit a possibly optimally-energy-distributed swimming style by hovering in the water layer and suddenly dashing. They either die if they fail to find more food bacteria, or they transform into trophonts if food bacteria are found. Gene enrichment analyses based on differentially expressed genes demonstrate that tomites are indeed in a dying state, as necessary physiological activities and cellular functions are significantly decreased and autophagy is up-regulated (Fig. 4D; Supplementary Table S3).

In conclusion, despite the crucial role in the ocean’s ecosystem, understanding the life-history evolution and response to environmental changes of marine ciliates has been a daunting task due to the lack of model organisms and research resources. However, this work provides a theoretical and technical framework for investigating the phenotypic plasticity of these tiny unicellular residents in coastal waters, and finds that phenotypic plasticity is preserved by purifying selection. By developing experimental and multi-omics resources, this study will help reveal molecular-level responses of non-model ciliates to various environmental changes. Also, the evolutionary strategies of these organisms may hold the key to their survival and evolution in the rapidly changing marine environment due to climate change. This knowledge offers new insights into the intricate workings of the inhabitants of the ocean ecosystem and the importance of phenotypic plasticity in the face of environmental change.

### Data access

All raw sequences in this research are publicly available at NCBI SRA under the BioProject Number of PRJNA860328. Two newly assembled and annotated *Glauconema* macronuclear genomes are uploaded to the National Genomics Data Center (https://www.cncb.ac.cn/), China National Center for Bioinformation: GWHDEDB00000000 (*Glauconema* sp1 LHA0827), GWHDEDC00000000 (*Glauconema* sp2 LJL43). All the scripts/input data for plotting and statistical analyses are in our lab’s GitHub (https://github.com/IEMB-LEG/PanJ_2024; the Zenodo DOI is https://zenodo.org/doi/10.5281/zenodo.12313062).

## Supplementary Material

Supplementary_Figures_for_Pan_et_al-0716_wrae136

Supplementary_Tables_for_Pan_et_al-0716_wrae136

Supplementary_Text_for_Pan_et_al-0716_wrae136
