# Supplementary material for: Molecular basis of phenotypic plasticity in a marine ciliate
Source: ISME J. 2024 Jul 17;18(1):wrae136. doi: 10.1093/ismejo/wrae136 (PMC11308186; doi:10.1093/ismejo/wrae136)
Supplement: Supplementary_Figures_for_Pan_et_al-0716_wrae136 [file supplementary_figures_for_pan_et_al-0716_wrae136.pdf]

Molecular basis of phenotypic plasticity in a marine ciliate

Jiao Pan[1,2,&], Yaohai Wang[1,&], Chao Li[1], Simo Zhang[3], Zhiqiang Ye[4], Jiahao Ni[1], Haichao Li[1], Yichen Li[1], Hongwei Yue[1], Chenchen Ruan[1], Dange Zhao[1], Yujian Jiang[1], Xiaolin Wu[1], Xiaopeng Shen[5], Rebecca A. Zufall[6], Yu Zhang[7], Weiyi Li[8], Michael Lynch[9], Hongan Long[*,1,2]

[1] Key Laboratory of Evolution and Marine Biodiversity (Ministry of Education), Institute of Evolution and Marine Biodiversity, Ocean University of China, Qingdao, Shandong Province, China 266003

[2] Laboratory for Marine Biology and Biotechnology, Qingdao Marine Science and Technology Center, Qingdao, Shandong Province, China 266237

[3] Department of Biology, Indiana University, Bloomington IN, USA 47405

[4] School of Life Sciences, Central China Normal University, Wuhan, Hubei Province, China 430079

[5] College of Life Sciences, Anhui Normal University, Wuhu, Anhui Province, China 241000

[6] Department of Biology and Biochemistry, University of Houston, Houston TX, USA 77204

[7] School of Mathematics Science, Ocean University of China, Qingdao, Shandong Province, China 266000

[8] Department of Genetics, Stanford University School of Medicine, Stanford CA, USA 94305

[9] Biodesign Center for Mechanisms of Evolution, Arizona State University, Tempe AZ, USA 85287

Short title: The phenotypic plasticity of *Glauconema*.

* Authors for correspondence:

Hongan Long, Key Laboratory of Evolution and Marine Biodiversity (Ministry of Education), Institute of Evolution and Marine Biodiversity, Ocean University of China, 5 Yushan Road， Qingdao, Shandong Province, China. Email: longhongan@ouc.edu.cn

& These authors contribute equally to this work.

Supplementary Figures

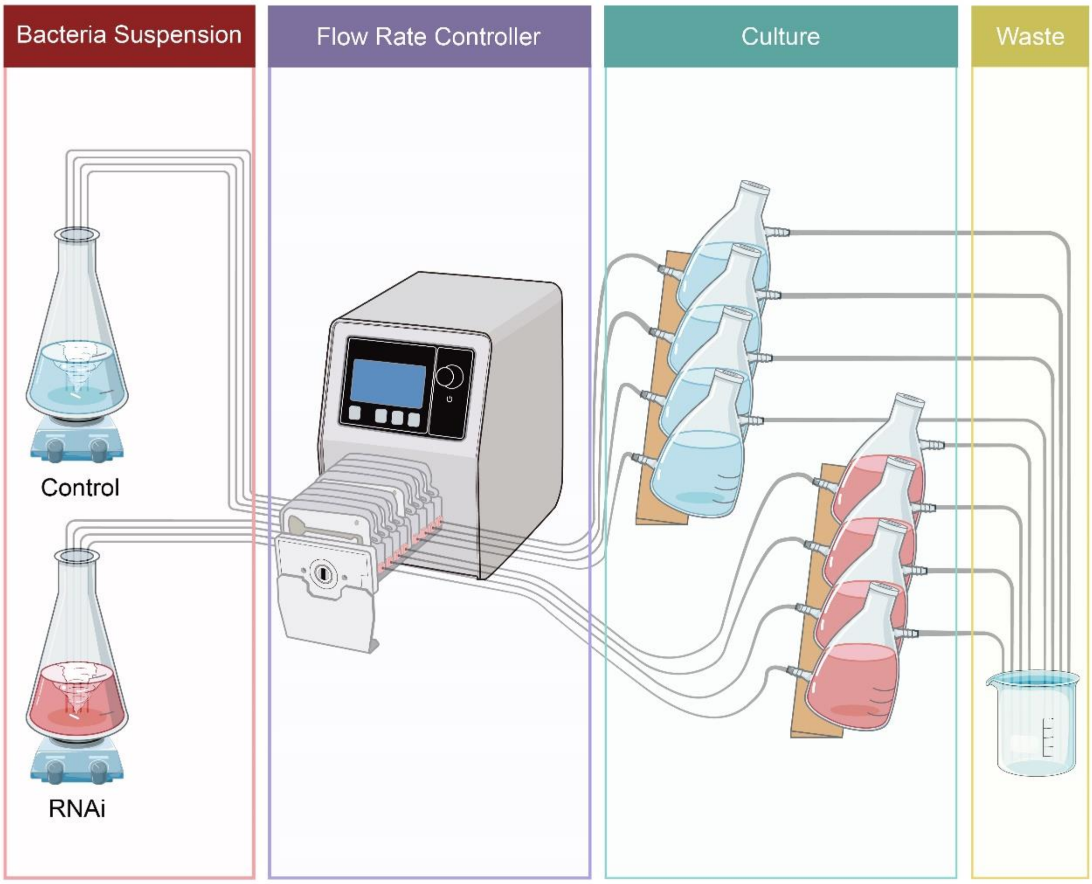

Supplementary Fig. S1. A lab-built chemostat-like culturing system. This device was used in both reaction norm derivation and RNAi experiments. Food bacteria densities in the leftmost flasks started from the same OD_{600} and kept at similar OD_{600} levels during the experimental span.

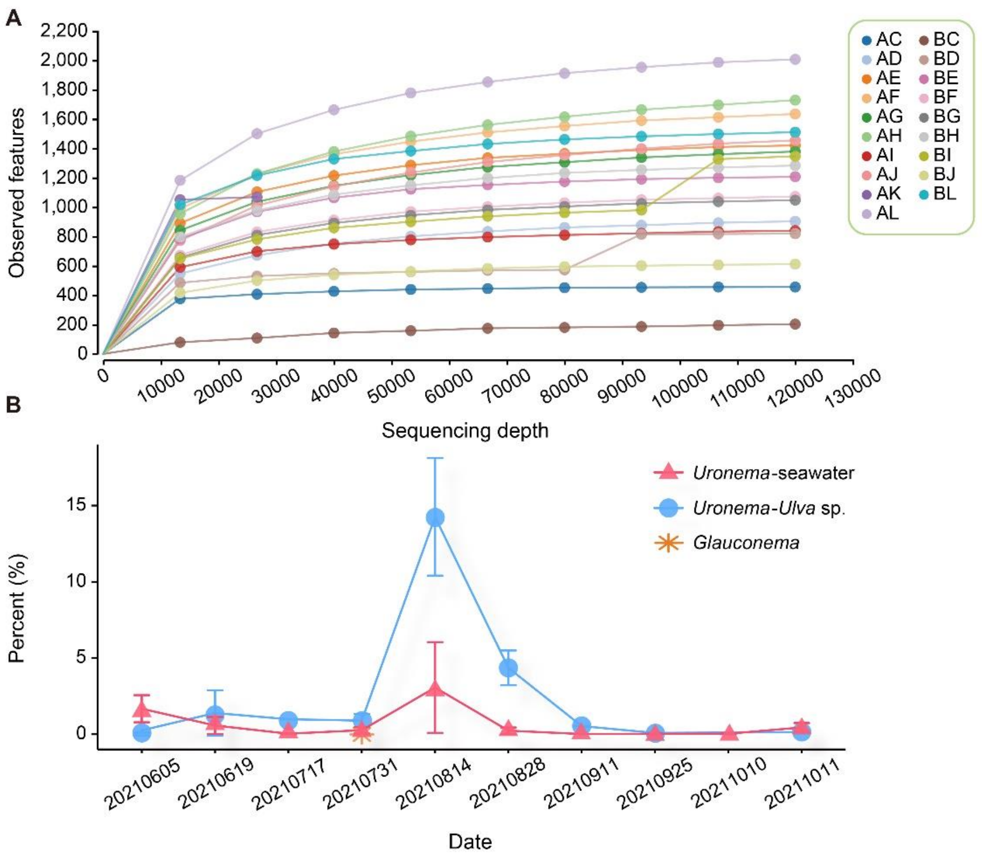

Supplementary Fig. S2. Community dynamics revealed by amplicon analyses. (A) The rarefaction curve of the number of feature sequences at different sampling time points. Rightmost legend shows the Sample IDs, more details are in Supplementary Table S2. **(B)** The percentages of amplicon reads of *Glauconema* and *Uronema* (out of all eukaryotic reads) from *Ulva* sp. or seawater at different sampling dates.

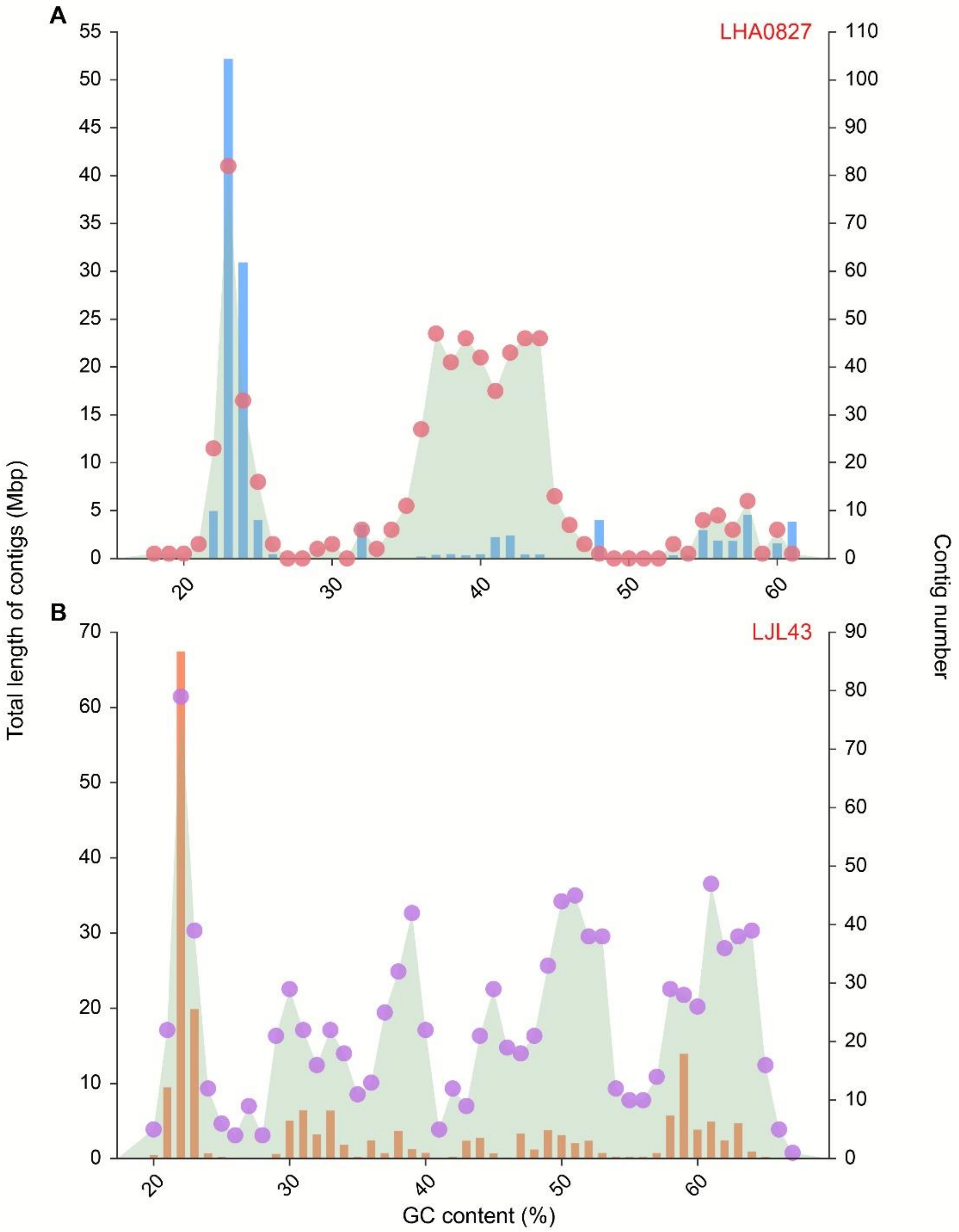

Supplementary Fig. S3. The distributions of GC content in macronuclear genomes of two *Glauconema* spp. before filtering (A: *G.* sp1 LHA0827; B: *G.* sp2 LJL43). X axis represents GC content; the left Y-axis (bar chart) represents the total length of contigs (Mbp) in each group. The right Y-axis (dot plot) represents the number of contigs.

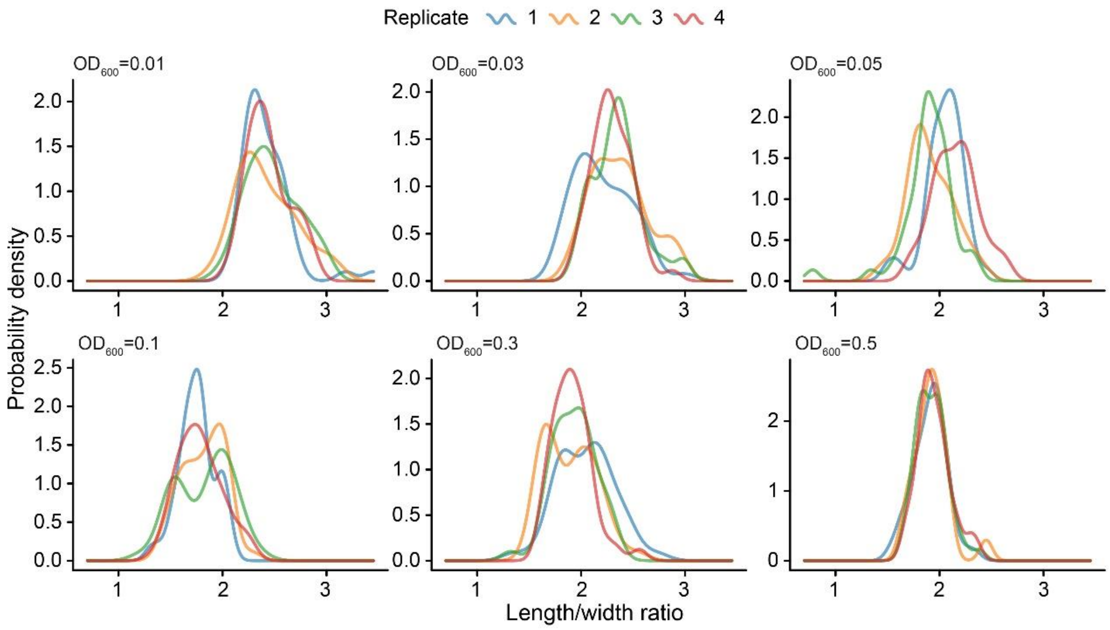

Supplementary Fig. S4. The distributions of the length/width ratio vs. bacterial density (measured in OD_{600}) of *Glauconema* sp1 LHA0827. Each curve represents data from an independent experiment conducted in a chemostat-like culturing system. Different colors represent different replicates.

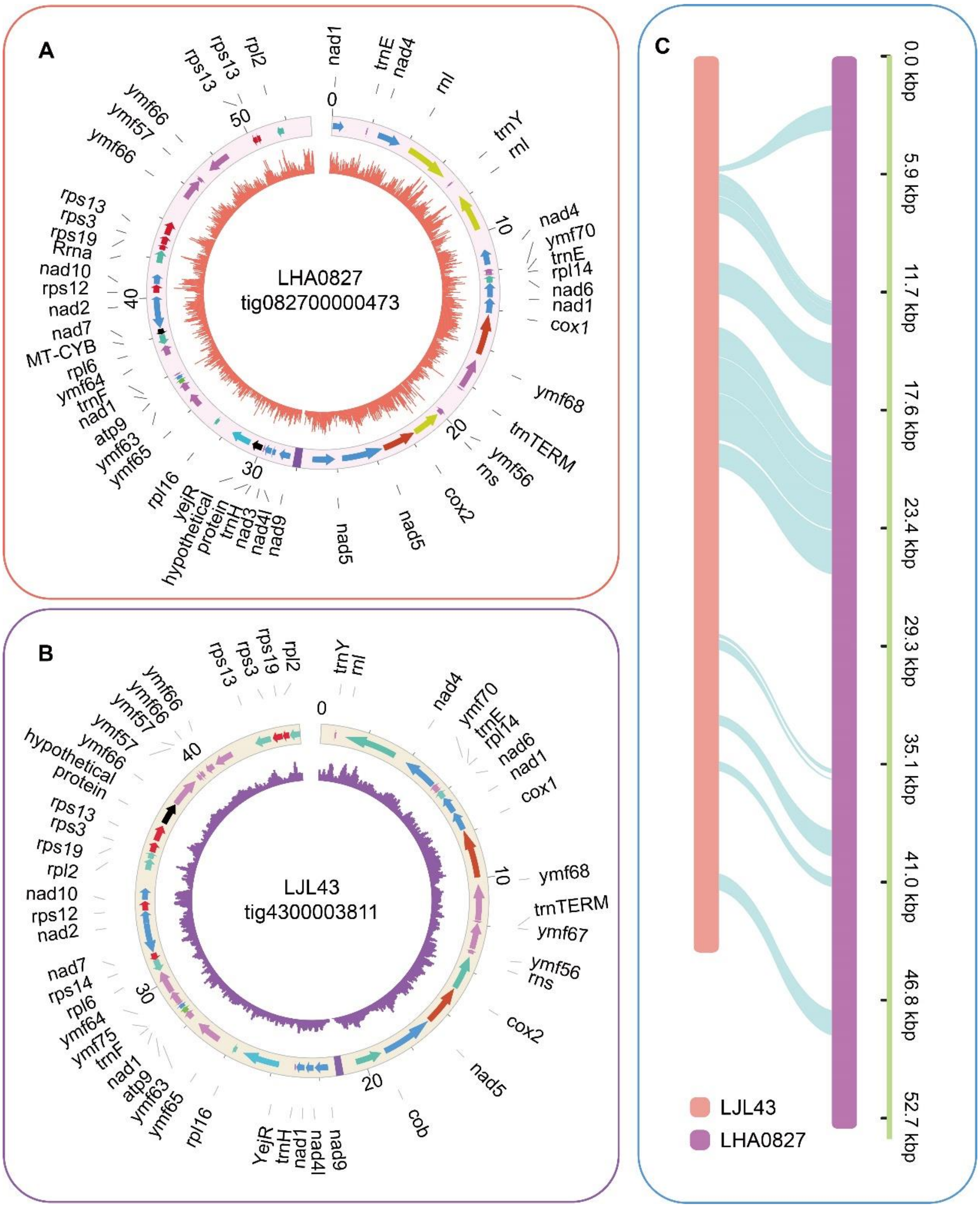

Supplementary Fig. S5. The assembly, annotation and co-linearity analyses of mitochondrial genomes of *Glauconema* spp. (A: *G.* sp1 LHA0827; B: *G.* sp2 LJL43). The numbers on the circle with arrows are in kbp; the arrow direction indicates the coding direction; the inner circle is the distribution of GC content. **(C)** The co-linearity of two *Glauconema* mitochondrial genomes.

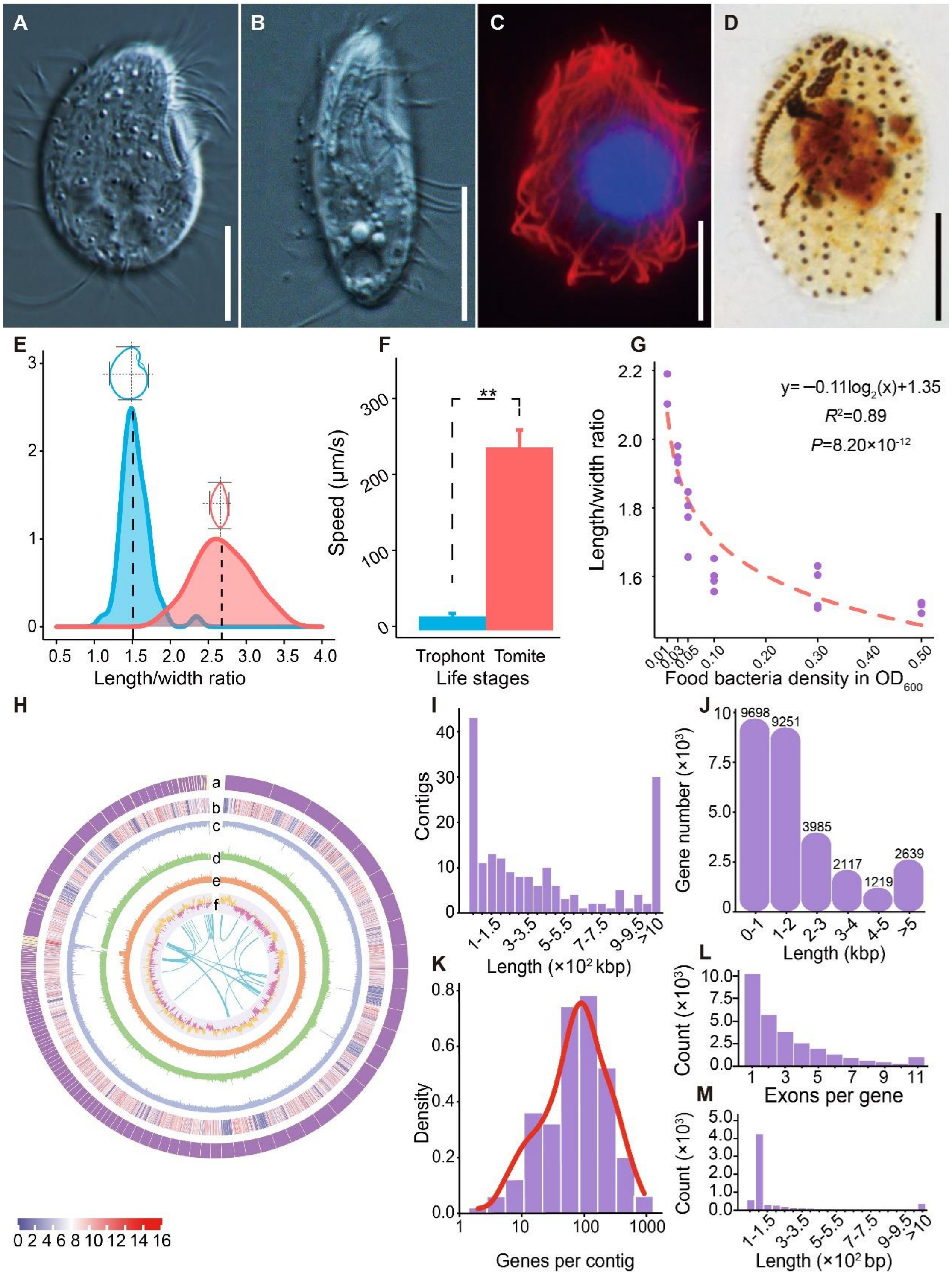

Supplementary Fig. S6. The phenotypic and genomic features of *Glauconema* sp2 LJL43. (A–D) The photomicrographs of living trophont (A) and tomite (B), after immunofluorescence staining (C), and protargol staining (D). **(E)** The length/width ratio distribution of trophonts (blue) and tomites (red). The black dashed lines represent the mean length/width ratio. **(F)** The swimming speed of trophonts and tomites. $^{**}P < 0.01$, based on Mann-Whitney *U* test. **(G)** The reaction norm of the length/width ratios vs. food bacteria density in OD_{600}, estimated in a chemostat-like culturing device. **(H)** Characteristics of the

assembled contigs of *G.* sp2 LJL43. a–f represent the contigs, the distribution of gene density (color legend on the up-left corner corresponds to this), genome coverage of nanopore reads, genome coverage of Illumina reads, GC density and GC skew with values calculated in 20 kbp sliding windows (5 kbp). The interconnecting lines represent collinear genes within the genome. **(I–M)** The distributions of contig length, gene length, gene number in each contig, exon number, and intron length.
