# Supplementary material for: Molecular basis of phenotypic plasticity in a marine ciliate
Source: ISME J. 2024 Jul 17;18(1):wrae136. doi: 10.1093/ismejo/wrae136 (PMC11308186; doi:10.1093/ismejo/wrae136)
Supplement: Supplementary_Text_for_Pan_et_al-0716_wrae136 [file supplementary_text_for_pan_et_al-0716_wrae136.pdf]

Procedures for sample collection to monitor the eukaryotic community dynamics in *Glauconema* habitats

Samples were immediately shipped to the lab on ice. For each sea lettuces sample, the tube was agitatedly vortexed for 5 min at top speed, and then filtered with gauze; cells in the control seawater samples were then enriched directly by centrifuging at 2000 g and 4 °C for 5 min. 71 samples in total were finally obtained. Cells in the filtrate were then centrifuged at 2000 g for 5 min at 4 °C. The MasterPure Complete DNA&RNA Purification kit (Cat. No.: MC85200; Lucigen, USA) was used to extract the total RNA of each sample. RNA was then reverse transcribed into cDNA, using the HiScript III 1st Strand cDNA Synthesis Kit (+gDNA wiper) (Cat. No.: R312; Vazyme, China). Primers (EukV8-9F 5'-ATAACTGGTCTGTGATGCCCT-3' and EukV8-9R 5'-CCTTCYGCAGGTTCACCTAC-3') were used to amplify the V8–V9 region of the 18S rRNA gene. Sequencing was performed on an NovaSeq 6000 System (Illumina), using 2×250 bp paired-end sequencing in accordance with the manufacturer's instructions, at LC SCIENCES (Hangzhou, China) and Novogene (Beijing, China).

16,809,885 PE250 reads were obtained and merged using FLASH ver. 2.2.00 [1]. fastp ver. 0.23.1 [2] was used for quality control of raw reads to be merged for high-quality clean tags. Chimeric sequences were filtered out using Vsearch ver. 2.18.0 [3]. After de-replication using DADA2 (--p-max-ee 2) in the QIIME2 ver. 2021.8 [4] and normalization of SILVA (release 138) classifier, the clusters formed 28,836 features of eukaryotes. Of these features, 6,845 could only be classified as eukaryotes.

Procedures for the macronuclear genome size prediction

To make sure that the sequenced *Glauconema* spp. genomes were assembled as "complete" as possible, we ran wtdbg2 and Flye with a series of genome sizes, by taking sizes of published ciliate macronuclear genomes into consideration: 100 Mbp, 120 Mbp, 200 Mbp, and 500 Mbp. For wtdbg2, as expected, with larger values of genome sizes, it generated longer assemblies. The increment in size slowed down from 200 Mbp to 500 Mbp (104,244,535 bp vs 106,723,632 bp), but the number of contigs assembled increased (470 vs 662). The majority of these additionally assembled sequences were presented as short contigs. Flye

yielded a similar pattern. The contigs obtained from the 500 Mbp run was even shorter than the one generated by giving a 200 Mbp value (114,319,181 vs 119,441,474), and the former had a much higher number of contigs (1,331 vs 340). We therefore chose 200 Mbp as the estimated genome size and ran Canu ver. 2.1.1 [5] assembler. All the assemblers were run with default parameters except for the expected genome sizes.

Details of phenotypic plasticity and *de novo* assembly of *Glauconema* sp2 LJL43

G. sp2 LJL43 also responds to food bacteria density similarly to *G.* sp1 LHA0827, with mean length/width ratios of trophonts and tomites of 1.54 and 2.69, respectively (*t*-test, $P = 4.48\times10^{-36}$; Supplementary Table S5; Supplementary Fig. S6A–S6E). Using the chemostat-like culturing system, we also derived the reaction norm for *G.* sp2 LJL43 ($BS = -0.11\times\log_2(FBD) + 1.35$, $R^2 = 0.89$, $P = 8.20\times10^{-12}$) (Supplementary Fig. S6G; Supplementary Table S11). There is a significant difference in the body shape of trophonts between *G.* sp2 LJL43 and *G.* sp1 LHA0827 (*t*-test, $P = 3.51\times10^{-16}$), but no difference for tomites (*t*-test, $P = 0.08$). Furthermore, the two congeners also differed in trophont swimming speed (9 in *G.* sp2 LJL43 vs. 31 μm/s in *G.* sp1 LHA0827; Mann-Whitney *U* test, $P = 3.11\times10^{-6}$), but not for tomite swimming speed (240 in *G.* sp2 LJL43 vs. 208 μm/s in *G.* sp1 LHA0827; Mann-Whitney *U* test, $P = 0.29$) (Supplementary Fig. S6F; Supplementary Table S6). The swimming trajectories of trophonts and tomites in *G.* sp2 LJL43 showed a similar pattern to that of *G.* sp1 LHA0827, with the tomite swimming area being much larger (Fig. 1J, 1K). The observation of the same tomite body shape and swimming speed in two species suggests that they are experiencing similar selective regimes for this particular body shape and swimming, or that the limit of phenotypic plasticity has been reached, meaning that tomites cannot become narrower or swim faster, although data from more species are needed to reach a conclusion.

To further investigate the evolution of *Glauconema* spp., we performed *de novo* assembly and annotation on *G.* sp2 LJL43 and obtained its macronuclear genome with a BUSCO score of 88%, totaling 98.87 Mbp in size. It consists of 181 contigs, with G/C content of 23%, N50 of 1.46 Mbp, and the longest contig of 4.08 Mbp. 96% of the contigs containing at least one telomere and 67% having two telomeres (Table 2; Supplementary

Table S8). We annotated 28,909 genes, with a mean gene size of 2.27 kbp. The mitochondrial genome size is 44,482 bp, encoding 43 complete protein-coding genes, 5 tRNAs and 2 rRNAs (Supplementary Fig. S5B; Supplementary Table S12). The two mitochondrial genomes revealed co-linearity between 14 pairs of genes (Supplementary Fig. S5C). The codon usages of both the macronuclear and mitochondrial genomes are the same as those of *G.* sp1 LHA0827 (Supplementary Table S9). Additional details about the genome are presented in Table 2, Supplementary Fig. S6H–S6M, and Supplementary Table S8.

References:

1. Magoč T, Salzberg SL. FLASH: fast length adjustment of short reads to improve genome assemblies. Bioinformatics. 2011;27(21):2957–63.
2. Chen S, Zhou Y, Chen Y, Gu J. fastp: an ultra-fast all-in-one FASTQ preprocessor. Bioinformatics. 2018;34(17):i884–i90.
3. Rognes T, Flouri T, Nichols B, Quince C, Mahé F. VSEARCH: a versatile open source tool for metagenomics. PeerJ. 2016;4:e2584.
4. Bolyen E, Rideout JR, Dillon MR, Bokulich NA, Abnet CC, Al-Ghalith GA, et al. Reproducible, interactive, scalable and extensible microbiome data science using QIIME 2. Nat Biotechnol. 2019;37(8):852–7.
5. Koren S, Walenz BP, Berlin K, Miller JR, Bergman NH, Phillippy AM. Canu: scalable and accurate long-read assembly via adaptive k-mer weighting and repeat separation. Genome Res. 2017;27(5):722–36.
